# Pterostilbene Attenuates Cocultured BV-2 Microglial Inflammation-Mediated SH-SY5Y Neuronal Oxidative Injury via SIRT-1 Signalling

**DOI:** 10.1155/2020/3986348

**Published:** 2020-08-04

**Authors:** Qiang Zhu, Tao Tang, Haixiao Liu, Yinxue Sun, Xiaogang Wang, Qiang Liu, Long Yang, Zhijie Lei, Zhao Huang, Zhao Chen, Qiang Lei, Mingyang Song, Bodong Wang

**Affiliations:** ^1^Department of Neurosurgery, The 960th hospital, 25th Shifan Road, Jinan, 250031 Shandong, China; ^2^Department of Neurosurgery, Yan'an University Affiliated Hospital, Yongxiang Road, Baota District, Yan'an, 716000 Shaanxi, China; ^3^Department of Neurosurgery, Tangdu Hospital, The Fourth Military Medical University, 1st Xinsi Road, Xi'an, 710038 Shaanxi, China; ^4^The 960th hospital, 25th Shifan Road, Jinan, 250031 Shandong, China; ^5^Department of Traditional Chinese Medicine, Shandong University of Traditional Chinese Medicine, Jinan, Shandong 250355, China

## Abstract

Microglial inflammation plays an important part in the progression of multiple neurological diseases, including neurodegenerative diseases, stroke, depression, and traumatic encephalopathy. Here, we aimed to explore the role of pterostilbene (PTE) in the microglial inflammatory response and subsequent damage of cocultured neural cells and partially explain the underlying mechanisms. In the coculture system of lipopolysaccharide-activated BV-2 microglia and SH-SY5Y neuroblastoma, PTE (only given to BV-2) exhibited protection on SH-SY5Y cells, evidenced by improved SH-SY5Y morphology and viability and LDH release. It also attenuated SH-SY5Y apoptosis and oxidative stress, evidenced by TUNEL and DCFH-DA staining, as well as MDA, SOD, and GSH levels. Moreover, PTE upregulated SIRT-1 expression and suppressed acetylation of NF-*κ*B p65 subunit in BV-2 microglia, thus decreasing the inflammatory factors, including TNF-*α* and IL-6. Furthermore, the effects above were reversed by SIRT-1 inhibitor EX527. These results suggest that PTE reduces the microglia-mediated inflammatory response and alleviates subsequent neuronal apoptosis and oxidative injury via increasing SIRT-1 expression and inhibiting the NF-*κ*B signalling pathway.

## 1. Introduction

Inflammation is considered to play a pivotal part in diverse diseases, including neurodegenerative diseases [1, 2], brain trauma [3], stroke [4], infection [5], and even mental disorders [6], in the central nervous system (CNS). Accumulating evidences suggest that microglia, the resident innate immune cells, which accounts for 10% of CNS cells, are the active participants in the pathophysiological processes related to these neuroinflammatory diseases [7]. In physiological situations, the microglia detect, transduce, integrate, and respond to extracellular signals, and participate in brain development and maintenance of CNS homeostasis by regulating programmed cell death, phagocytosis, and synaptic plasticity [7]. However, microglia dysfunction in pathological conditions and promote neurotoxicity through excessive inflammatory cytokine release [8]. Studies have shown that microglia-mediated inflammation and production of proinflammatory factors, including interleukins (ILs) and tumour necrosis factor (TNF) or noninflammatory factors, such as superoxide ions, are important pathogenic bases of neurological diseases, such as intracranial infections, stroke, trauma, and Alzheimer's disease (AD) [1–6]. These overly released superoxide and cytokines trigger the oxidative injury and apoptosis of neurons. Therefore, targeting microglial functions and regulating microglia-mediated inflammatory processes and oxidative stress may yield potent paradigms for therapies of CNS disorders that were inconceivable under a neuron-centric view of the brain [7].

Pterostilbene (PTE), also known as trans-3,5-dimethoxy-4′-hydroxystilbene, is a natural stilbenoid in grapes, berries, and Chinese herbs. It belongs to the dimethylated analogs of resveratrol and is well known for its indisputable anti-inflammatory activity in vitro and in vivo [9, 10]. Moreover, PTE exhibit many pharmacological activities [11], including antiaging [12], anticancer [13, 14], antidiabetes [15], regulating fat metabolism [15–17], antioxidation [18–20], antidepression [10], and neuroprotection [10, 21]. Intriguingly, in the CNS, PTE are able to penetrate the blood brain barrier (BBB) and to modulate neural activity [22], which may exert protection against degenerative disorders [21], hyperprolactinemia [23], and stroke [18, 24, 25]. The biological effects of PTE on neural cells involve the regulation of neurogenesis [10], apoptosis [19], neuroinflammation [18, 24], and oxidative stress [19, 20]. Previously, we have found that PTE attenuates neural impairments in different neurological disorders, including glutamate or high-glucose-induced oxidative stress in neural cells [19, 20], inflammation and mitochondrial oxidative stress injury after cerebral ischemia reperfusion [18, 25], inflammation and oxidation-involved early brain injury following subarachnoid haemorrhage (SAH) [24]. Additionally, recent studies have shown that PTE inhibits the amyloid-*β*- and lipopolysaccharide- (LPS-) induced activation of microglia [26–28], whereas, it remains not fully clear whether PTE exerts neuronal protective effects by regulating microglia-mediated inflammatory and oxidative injury, as well as the molecular mechanisms.

Sirtuin 1 (SIRT-1), one of the seven mammalian homologues belonging to the silent mating type information regulation 2 family, is a regulator of proteins and genes involved in antioxidation, anti-inflammation, antiapoptosis, insulin response, metabolism, mitochondrial biogenesis, synaptic plasticity, stress resistance, and genomic stability, which is thus important in cell survival under stressful conditions [29, 30]. In the CNS, SIRT-1 plays an important role in promoting neurodevelopment, delaying brain senescence, maintaining homeostasis, and modulating circadian rhythm [30, 31] and has also been demonstrated as the neuroprotective roles under the condition of neurodegeneration and cerebral ischemia [30]. Furthermore, accumulating studies have suggested that SIRT-1 exhibits a key role in regulating neuroinflammation following CNS disturbances via inhibiting NACHT domain-, leucine-rich repeat-, and PYD-containing protein 3 (NLRP3) inflammasome activation, Toll-like receptor (TLR) 4 signalling, nuclear factor- (NF-) *κ*B pathway, and IL-1*β* transcription, which may relate to the modulating of microglial function [32–35]. Previously, we have also found that SIRT-1/NF-*κ*B signalling could attenuate inflammatory injury in experimental SAH models [36]. Interestingly, PTE has been proved as a potent SIRT-1 activator in different cells, including hepatic cells [37], cardiomyocytes [38], and skeletal muscle cells [39]. However, the role of SIRT-1 signalling in microglia after PTE treatment and the relationship between microglial inflammation and neuronal oxidative injury in the PTE mediated neuroprotection have not been well investigated.

In view of the above, PTE presents the therapeutic potential to prevent or reduce microglial inflammation and neuronal oxidative stress injury in neurological disorders, such as neurodegenerative diseases, trauma, brain stroke, and infections. Therefore, in this study, we aimed to evaluate a possible modulating activity of PTE on the microglia-mediated inflammatory and neuronal oxidative injuries and to explore the underlying mechanisms mediated by SIRT-1signalling.

## 2. Material and Methods

### 2.1. Materials

PTE, EX527, and DCFH-DA and DAPI fluorescent probes were obtained from Sigma-Aldrich (St. Louis, MO, USA). Lactate dehydrogenase (LDH) cytotoxicity, Cell counting kit-8 (CCK-8), MDA, SOD, and GSH assay kits were obtained from Beyotime Biotechnology (Shanghai, China). TUNEL kit was obtained from Roche (Mannheim, Germany). TNF-*α* and IL-6 ELISA kits were obtained from Sangon Biotech (Shanghai, China). Antibodies against SIRT-1, p65, and acetylated p65 at Lys310 were obtained from Cell Signalling Technology (Danvers, MA, USA). Antibodies against *β*-actin, caspase 3, cleaved-caspase 3, Bax, Bcl-2, and secondary antibodies labelled by HRP were obtained from Wanleibio (Shenyang, China). Transwell Chamber was purchased from Corning (NY, USA). Cell culture reagents were obtained from Gibco (Grand Island, NY, USA). SH-SY5Y human neuroblastoma cell line and BV-2 mouse microglia cell line were obtained from the Neurological Lab of Tangdu Hospital.

### 2.2. Experimental Protocol

- Step 1. Evaluate the influences of PTE on the BV-2/SH-SY5Y cocultivation system, containing the effects on SH-SY5Y survival and SIRT-1 expression within BV-2 cells. The cells were randomly divided into the control and PTE (2.5, 5.0, or 10.0 *μ*M) groups (*n* = 6). The groups were incubated with PTE or vehicle (0.01% DMSO) for 2 h and with FBS-free DMEM for another 24 h, followed by detections
- Step 2. Investigate whether PTE treatment alleviates the injury of SH-SY5Y cells induced by LPS-activated BV-2 cells. Firstly, the damage effect on SH-SY5Y cells of LPS-activated BV-2 microglia was verified. The SH-SY5Y neuroblastomas were randomly divided into the control, BV-2 alone, LPS alone, and LPS-activated BV-2 groups (*n* = 6), which were separately incubated or cocultured with the vehicle, BV-2 cells, LPS (100 ng/mL), or BV-2 cells with LPS (100 ng/mL) stimulation for 24 h. Secondly, the protective effects of PTE were tested. The SH-SY5Y cells were randomly divided into the control, LPS-activated BV-2 cocultured, LPS-activated BV-2 cocultured+PTE (2.5, 5.0, or, 10.0 *μ*M)-treated groups (*n* = 6). In the experimental groups, the BV-2 microglia with the stimulation of LPS (100 ng/mL) were pretreated with PTE or vehicle for 2 h, followed by coculturing with SH-SY5Y cells for 24 h. The BV-2 microglia of the control group were treated with vehicle and without LPS stimulation. After interventions above, further detections were carried out, including cell survival, apoptosis, oxidative stress, and inflammatory factor.
- Step 3. Explore the role of SIRT-1 in the protective effect of PTE against the injury induced by the LPS-activated BV-2 microglia. The SH-SY5Y cells were randomly divided into the control, LPS-activated BV-2 cocultured, LPS-activated BV-2 cocultured+5*μ*M PTE, LPS-activated BV-2 cocultured+5 *μ*M PTE+EX527 groups (*n* = 6). The BV-2 cells in the EX527-treated group were preincubated with 100 nM EX527 for 24 h, and other interventions of all groups were the same as step 2. After the interventions, further detections were carried out, including the expression of proteins, cell survival, apoptosis, oxidative stress, and inflammatory factor.

### 2.3. Cell Culture and Treatments

SH-SY5Y and BV-2 cells were cultured with Dulbecco's modified Eagle medium (DMEM) as previously described [18]. Culture media were changed every 2 days. PTE and EX527 were dissolved with dimethylsulfoxide (DMSO) and then diluted in DMEM before experiments (DMSO ≤ 0.1%). The coculture system was established as previously described [40]. In the coculture assay, BV-2 cells were cultured in a Transwell chamber (pore size 0.4 *μ*m, polylysine-coated polycarbonate membrane) at a density of 2 × 10^5^/well, and then treated with PTE or vehicle for 2 h, followed by LPS (diluted with phenol red-free DMEM at a final concentration of 100 ng/mL) stimulation and coculturing with SH-SY5Y cells for 24 h. In the inhibitory assay, BV-2 microglia were preincubated with EX527 at a final concentration of 100 nM.

### 2.4. Cell Viability and LDH Release Assay

The cell viability and LDH generation were measured using the CCK-8 and LDH release assay kits according to the manual. Briefly, following experimental intervention, cells were incubated with CCK-8 solution for 3 h at 37°C, and then the supernatants were transferred into a 96-well plate and detected at 450 nm. To detect LDH level, the culture media were transferred and incubated with 60 *μ*L LDH detection buffer per well in a 96-well plate on a constant temperature (25°C) lightproof shaker for 30 min. Finally, the absorbance was detected using the microplate reader at 490 nm. The percentage of LDH release was then computed.

### 2.5. Western Blotting Assay

Cells were harvested after experimental intervention and lysed in 200 *μ*L lysis buffer. Protein quantification and western blotting were conducted as previously described [18]. In short, the prepared samples were separated on the SDS-PAGE gel and then transferred onto a PVDF membrane, which was then cut into stripes according to the markers and incubated with different primary antibodies for 12 h at 4°C. Next, the stripes were washed and incubated with corresponding secondary antibodies for 2 h at 25°C. The concentrations of different antibodies were prepared as follows: anti-SIRT-1 (1 : 1000), anti-Ac-p65 (1 : 1000), anti-p65 (1 : 1000), anti-*β*-actin (1 : 2000), anti-Bcl-2 (1 : 1000), anti-Bax (1 : 1000), anti-cleaved-caspase 3 (1 : 500), anti-caspase 3 (1 : 500), and secondary HRP-labelled antibodies (1 : 20000). Finally, the stripes were reacted with a chemiluminescent reagent and then scanned under an imaging system (Bio-Rad, Hercules, CA), followed by analysis using ImageJ software (version 1.46).

### 2.6. TUNEL Staining

Cells were cultured in a special 24-well plate used for fluorescent detection. After the experimental intervention, cells were fixed in 4% paraformaldehyde for 30 min, and then reacted with a TUNEL solution for 1 h, and subsequently incubated with DAPI for 15 min at 37°C, followed by a fluorescence microscopy. Images were analysed using ImageJ software (version 1.46).

### 2.7. DCFH-DA Staining

The intracellular reactive oxygen species (ROS) were stained using fluorescent probe DCFH-DA as previously described. After the experimental intervention, the cells were treated with 10 *μ*mol/L DCFH-DA in a lightproof place at 37°C for 30 min, followed by a fluorescence microscopy. Images were analysed using ImageJ software (version 1.46).

### 2.8. Measurement of MDA, SOD, GSH, TNF-*α*, and IL-6

Generally, after the experimental intervention, the cell samples were lysed and centrifuged to obtain intracellular supernatants. The MDA level, SOD activity, GSH level, and TNF-*α* and IL-6 levels in the supernatants were assessed using corresponding commercial detection kits, according to the manufacturer's instructions.

### 2.9. Statistical Analysis

All data were analysed using GraphPad Prism 5.0 (GraphPad Software Inc., La Jolla, CA) and are shown as mean ± standard error of mean (SEM). Group means were compared by one-way analysis of variance (ANOVA), and Tukey's post hoc tests were then performed for significant groups. It was considered significant when *p* < 0.05.

## 3. Results

### 3.1. The Effects of PTE on Cell Viability of SH-SY5Y, LDH Release, and SIRT-1 Expression in BV-2 Cells, in Coculture System

**Figure 1:**
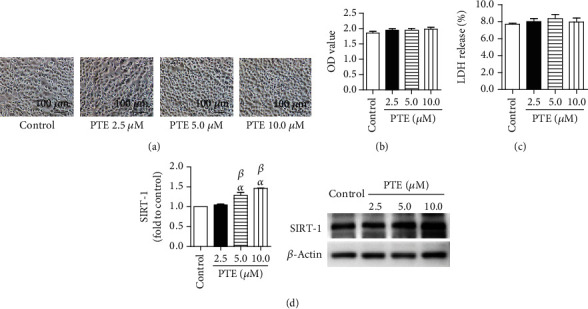
Effects of PTE on the SH-SY5Y and BV-2 coculture systems. The BV-2 cells were incubated with vehicle control or PTE (2.5, 5.0, or 10.0 *μ*M) for 2 h, followed by cocultivation with SH-SY5Y cells for 24 h. (a) The morphology of SH-SY5Y cells was photographed under an inverted/phase-contrast microscope, and (b) viability was quantified using a CCK-8 assay. (c) The LDH release in supernatants was determined using an LDH release assay and expressed as the percentage of maximum. (d) The expression of SIRT-1 in BV-2 cells was measured using western blot analysis and normalized to that of *β*-actin. Data are shown as mean ± SEM. *n* = 6. ^*α*^*p* < 0.05, compared with the control. ^*β*^*p* < 0.05, compared with PTE 2.5 *μ*M.

We first investigated the effects of PTE on the SH-SY5Y and BV-2 coculture systems without LPS stimulation at different concentrations. PTE (2.5, 5.0, or 10.0 *μ*M) treatment had no effects on SH-SY5Y cell viability or LDH release in the cocultured system (Figures 1(a)–1(c)). Interestingly, PTE treatment (5.0 and 10.0 *μ*M) significantly increased SIRT-1 expression in BV-2 cells to 1.29 ± 0.08- and 1.46 ± 0.01-fold, respectively (Figure 1(d)).

### 3.2. The Effects of PTE on Cell Viability, LDH Release, and Apoptotic Rate of SH-SY5Y in LPS-Activated BV-2 Coculture System

**Figure 2:**
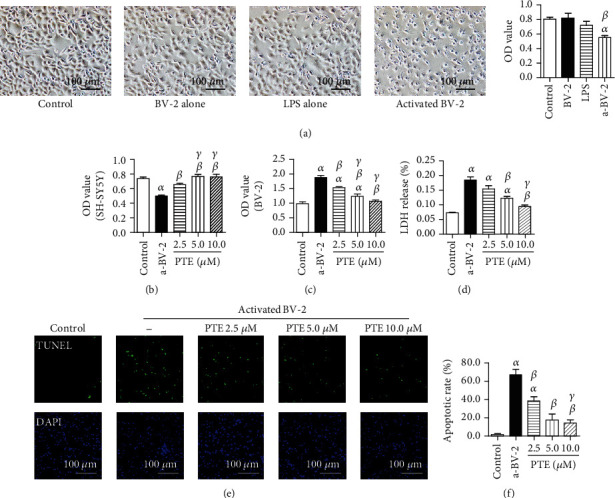
LPS induces BV-2 activation and SH-SY5Y injury in the coculture system, and PTE shows protective effects after LPS stimulation. SH-SY5Y cells were cocultured with BV-2 cells, LPS (100 ng/mL), or both BV-2 cells and LPS for 24 h, separately. (a) The morphology and viability of SH-SY5Y cells were observed under an inverted/phase-contrast microscope or using a CCK-8 assay, respectively. The BV-2 cells were preincubated with vehicle control or PTE (2.5, 5.0, or 10.0 *μ*M) for 2 h, followed by LPS stimulation and coculturing with SH-SY5Y cells for 24 h. The viability of (b) SH-SY5Y and (c) BV-2 cells was measured using a CCK-8 assay. (d) The LDH release in supernatants was determined using an LDH release assay and expressed as the percentage of maximum. (e) The apoptosis of SH-SY5Y cells was assessed using a TUNEL assay. The apoptotic nuclei were stained with TUNEL (green), and all nuclei were stained with DAPI (blue). (f) Apoptotic rate was computed as a percentage of the TUNEL- to the DAPI-positive nuclei. Data are shown as mean ± SEM. *n* = 6. ^*α*^*p* < 0.05, compared with the control. ^*β*^*p* < 0.05, compared with the a-BV-2. ^*γ*^*p* < 0.05, compared with the PTE 2.5 *μ*M. LPS: lipopolysaccharide; a-BV-2: LPS-activated BV-2 coculture group.

As shown in Figure 2(a), SH-SY5Y cells were cultured with the vehicle, BV-2 cells, LPS, and LPS-activated BV-2 cells for 24 h. Either BV-2 or LPS alone had no effect on cell morphology and viability of SH-SY5Y. Obviously, LPS-activated BV-2 microglia shrank and floated cocultured SH-SY5Y cells in morphology and weakened the viability of SH-SY5Y cells (OD value = 0.81 ± 0.03) compared with that of the control (OD value = 0.55 ± 0.03). In the cocultured system, the OD values of SH-SY5Y and BV-2 in the control group were 0.74 ± 0.02 and 0.99 ± 0.06, and LPS stimulation impaired SH-SY5Y viability (OD value = 0.50 ± 0.01) and promotes BV-2 viability (OD value = 1.88 ± 0.07). PTE (2.5, 5.0, or 10.0 *μ*M) significantly increased OD values of SH-SY5Y to 0.65 ± 0.02, 0.76 ± 0.03, and 0.76 ± 0.04 and decreased those of BV-2 cells to 1.54 ± 0.03, 1.24 ± 0.08, and 1.07 ± 0.04, respectively (Figures 2(b) and 2(c)). Similarly, PTE (2.5, 5.0, or 10.0 *μ*M) decreased LDH release in supernatant to 0.15 ± 0.01%, 0.12 ± 0.01%, and 0.09 ± 0.00% compared with 0.18 ± 0.01% of the LPS-activated BV-2 group (Figure 2(d)). In addition, apoptotic SH-SY5Y were stained with TUNEL. The apoptosis rate in LPS-activated BV-2 coculture group were 67.10 ± 6.08%, and PTE (2.5, 5.0, or 10.0 *μ*M) treatment significantly decreased that to 38.6 ± 4.51%, 17.23 ± 6.96%, and 14.01 ± 3.83%, respectively (Figures 2(e) and 2(f)).

### 3.3. The Effects of PTE on Oxidative Stress of SH-SY5Y and Inflammatory Factors in Supernatant in LPS-Activated BV-2 Coculture System

**Figure 3:**
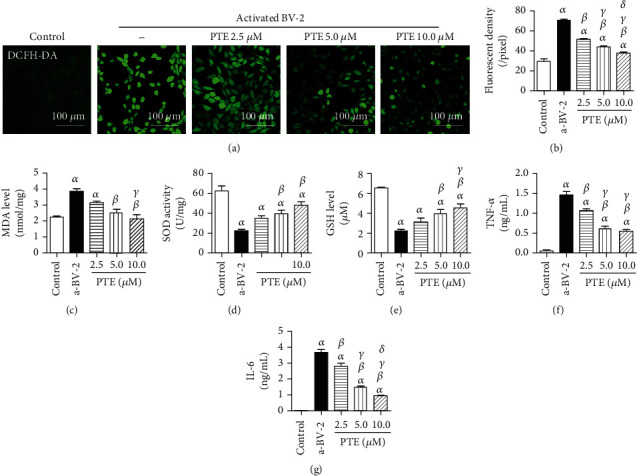
PTE reduces inflammatory factors released by LPS-activated BV-2 cells and attenuates the oxidative stress level of SH-SY5Y cells. BV-2 cells were pretreated with vehicle control or PTE (2.5, 5.0, or 10.0 *μ*M) for 2 h, followed by LPS stimulation and coculturing with SH-SY5Y cells for 24 h. (a) The ROS in SH-SY5Y cells was stained with DCFH-DA (green), and (b) the level of ROS was computed as the average intracellular fluorescent density. (c) The MDA level, (d) SOD activity, and (e) GSH level in SH-SY5Y cells were assessed using standardized commercial kits, and the (f) TNF-*α* and (g) IL-6 in supernatants were determined using an ELISA assay. Data are shown as mean ± SEM. *n* = 6. ^*α*^*p* < 0.05, compared with the control. ^*β*^*p* < 0.05, compared with the a-BV-2. ^*γ*^*p* < 0.05, compared with the PTE 2.5 *μ*M. ^*δ*^*p* < 0.05, compared with the PTE 5.0 *μ*M. LPS: lipopolysaccharide; a-BV-2: LPS-activated BV-2 coculture group; ROS: reactive oxygen species; MDA: malondialdehyde; SOD: superoxide dismutase; GSH: glutathione.

DCFH-DA staining was used to mark oxidative SH-SY5Y cells, the average intracellular fluorescent density were 29.48 ± 2.45 per pixel in the control group and significantly increased to 70.76 ± 1.03 per pixel in the LPS-activated BV-2 coculture group. PTE (2.5, 5.0, or 10.0 *μ*M) treatment decreased the fluorescent density to 51.31 ± 0.85, 43.63 ± 1.43, and 37.81 ± 1.09 in a dose-dependent manner (Figures 3(a) and 3(b)). Similarly, PTE treatment decreased the MDA level, increased the SOD activity, and elevated the GSH level in SH-SY5Y cells, compared with those of the LPS-activated BV-2 coculture group (3.83 ± 0.19 mmol/mg, 22.84 ± 0.99 U/mg, and 2.23 ± 0.15 *μ*M). These effects were significant with 5.0 *μ*M and 10.0 *μ*M PTE, which separately changed the MDA level, SOD activity, and GSH level to 2.51 ± 0.23 mmol/mg, 39.69 ± 3.33 U/mg, and 3.94 ± 0.39 *μ*M at 5.0 *μ*M and 2.10 ± 0.29 mmol/mg, 48.05 ± 3.65 U/mg, and 4.59 ± 0.37 *μ*M at 10.0 *μ*M, respectively (Figures 3(c)–3(e)).

The levels of inflammatory factors, TNF-*α* and IL-6, in the supernatant were 1.46 ± 0.09 ng/mL and 3.70 ± 0.16 ng/mL in the LPS-activated BV-2 coculture group, compared with 0.08 ± 0.00 ng/mL and 0.01 ± 0.01 ng/mL in the coculture group without LPS stimulation. PTE (2.5, 5.0, or 10.0 *μ*M) separately decreased the levels of TNF-*α* to 1.07 ± 0.04 ng/mL, 0.61 ± 0.06 ng/mL, and 0.55 ± 0.04 ng/mL and those of IL-6 to 2.81 ± 0.20 ng/mL, 1.49 ± 0.07 ng/mL, and 0.95 ± 0.03 ng/mL, in a dose-dependent manner (Figures 3(f) and 3(g)).

### 3.4. The Effects of PTE and EX527 on Expression of SIRT-1 and Acetylated p65 in LPS-Activated BV-2 Microglia and Inflammatory Factors in Supernatant

**Figure 4:**
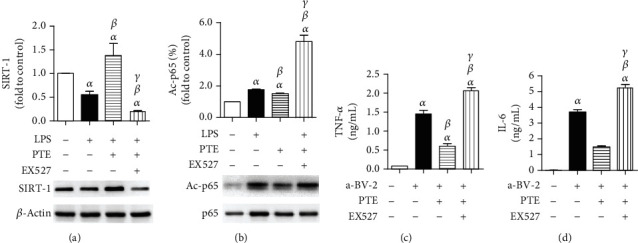
PTE promotes SIRT-1 expression, suppresses acetylation of p65 unit of NF-*κ*B, and reduces inflammatory factor release in LPS-activated BV-2 cells, which can be abolished by EX527. BV-2 microglial cells were pretreated with vehicle or EX527 (100 nM) for 24 h and then incubated with vehicle or PTE (5.0 *μ*M) for 2 h, followed by coculturing with SH-SY5Y cells with or without the presence of LPS (100 ng/mL) for 24 h. (a) The expression of SIRT-1 in BV-2 cells was measured using western blot analysis and normalized to that of *β*-actin. (b) The acetylated p65 (L310) in BV-2 cells was measured using western blot analysis and normalized to that of total p65. (c) The TNF-*α* and (d) IL-6 in supernatants were determined using an ELISA assay. Data are shown as mean ± SEM. *n* = 6. ^*α*^*p* < 0.05, compared with the control. ^*β*^*p* < 0.05, compared with the a-BV-2. ^*γ*^*p* < 0.05, compared with the PTE 2.5 *μ*M. LPS: lipopolysaccharide; a-BV-2: LPS-activated BV-2 coculture group.

We further estimated the expression of SIRT-1 and acetylated NF-*κ*B subunit p65 in BV-2 microglia. LPS stimulation suppressed the expression of SIRT-1 and promoted the acetylation of p65, and PTE treatment significantly increased the level of SIRT-1 and decreased acetylated p65. However, EX527 treatment obviously reversed the effects of PTE (Figures 4(a) and 4(b)). In addition, The levels of TNF-*α* and IL-6 in the supernatant were increased in the LPS-activated BV-2 coculture group, and then those were decreased in 5.0 *μ*M PTE-treated group, while the effects of PTE were significantly reversed in the EX527-treated group, which increased the levels of TNF-*α* and IL-6 to 2.07 ± 0.07 ng/mL and 5.28 ± 0.18 ng/mL (Figures 4(c) and 4(d)).

### 3.5. The Effects of PTE and EX527 on Apoptosis and Oxidative Stress of SH-SY5Y in LPS-Activated BV-2 Coculture System

**Figure 5:**
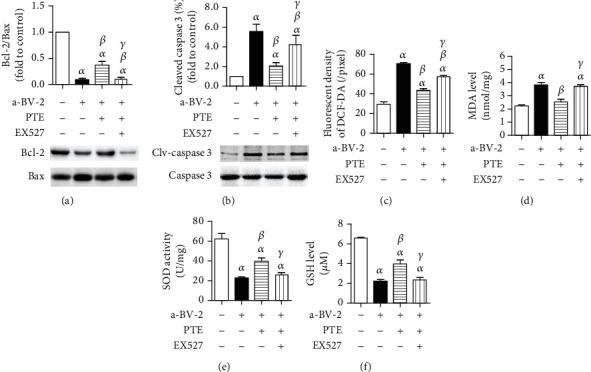
PTE attenuates apoptosis and oxidative stress of SH-SY5Y cells in the cocultured system with LPS-activated BV-2 cells, which can be abolished by EX527. BV-2 cells were pretreated with vehicle or EX527 (100 nM) for 24 h and then incubated with vehicle or PTE (5.0 *μ*M) for 2 h, followed by coculturing with SH-SY5Y cells with or without the presence of LPS (100 ng/mL) for 24 h. (a) The expression of Bcl-2 and Bax in SH-SY5Y cells was measured using western blot analysis and was shown as the ratio of Bcl-2 to Bax. (b) The cleaved caspase 3 in SH-SY5Y cells was measured using western blot analysis and normalized to that of total caspase 3. (c) The level of ROS was measured using DCFH-DA staining and expressed as an average intracellular fluorescent density. (d) The MDA level, (d) SOD activity, and (e) GSH level in SH-SY5Y cells were measured using standardized commercial kits. Data are shown as mean ± SEM. *n* = 6. ^*α*^*p* < 0.05, compared with the control. ^*β*^*p* < 0.05, compared with the a-BV-2. ^*γ*^*p* < 0.05, compared with the PTE 2.5 *μ*M. LPS: lipopolysaccharide; a-BV-2: LPS-activated BV-2 coculture group; ROS: reactive oxygen species; MDA, malondialdehyde: SOD, superoxide dismutase; GSH; glutathione.

As shown in Figures 5(a) and 5(b), LPS stimulation on BV-2 cells significantly decreased the ratio of Bcl-2 to Bax and increased the percentage of cleaved-caspase 3 in cocultured SH-SY5Y cells, compared with those in the control group, and PTE (5.0 *μ*M) treatment significantly reversed these effects. However, EX527 abolished the effects of PTE. PTE also decreased the fluorescent density of the DCFH-DA and MDA levels and increased the SOD activity and GSH level of SH-SY5Y cells, compared with those of the LPS-activated BV-2 coculture group. These effects of PTE were obviously abolished by EX527, which increased the fluorescent density of the DCFH-DA and MDA levels to 57.22 ± 1.28 per pixel and 3.72 ± 0.12 nmol/mg and decreased the SOD activity and GSH level to 25.79 ± 2.48 U/mg and 2.36 ± 0.23 *μ*M, respectively (Figures 5(c)–5(f)).

## 4. Discussion

PTE is a stilbenoid presenting in grapes and berries and also the biologically active compound of a Chinese herb, dragon's blood [10]. PTE has biological activities penetrating the BBB and shows no toxicity to neurons [22, 41]. Previously, we reported that PTE shows no toxic effect on HT22 neural cells at 10 *μ*M in vitro [19]. In this study, 10.0 *μ*M PTE treatment had no effects on SH-SY5Y cell viability or LDH release in the cocultured system, and this is consistent with the observation reported.

Recently, accumulating evidences have demonstrated the benefits of PTE and other analogue stilbenoids, including resveratrol, piceatannol, and gnetol, against inflammatory and oxidative processes in vitro and in vivo [9]. The anti-inflammatory effects of PTE involve many intra- and extracellular signals, including nitric oxide (NO), inducible nitric oxide synthase (iNOS), NLRP3, NF-*κ*B, TNF-*α*, and ILs [26–28, 42, 43]. We previously reported that PTE alleviates early inflammatory injury after SAH by inhibiting NLRP3 inflammasome and Nox2-related oxidative stress [24] and also attenuates the secondary astrocyte-induced inflammatory and oxidative stress after ischemia reperfusion injury by suppressing NF-*κ*B signalling [18]. This anti-inflammatory activity together with the antioxidant activity of PTE is believed to stand behind the neuroprotective effects against neurological disorders, especially the neurodegenerative diseases [21, 26, 27, 41, 42, 44] and stroke [39]. However, the effector cells and the underlying mechanisms are not entirely elucidated.

Microglia were first properly visualized and described as a distinct cell population in 1919 and have been considered the resident phagocytes of the innate immune system in the CNS since 1924. It was until the 1980s did researchers found that the microglia act as the indicator of immune activation and are correlated with the amyloid plaques in AD [45]. Multiple studies suggested that the microglia play an important role in the CNS health and disease, involving the surveillance of local environment, phagocytosis, releases of cytokines, chemokines, and growth factors, as well as the interaction with infiltrating immune cells [46]. More recently, studies have indicated that the microglia drives programmed cell death by inducing apoptosis of neurons through the release of superoxide ions, nerve growth factor, or TNF, without provoking inflammation, as well as prune developing axons and synapses and regulates neuronal and synaptic plasticity [7]. With the technical advances, the proinflammatory M1-like and anti-inflammatory M2-like polarization phenotypes of the microglia were found, although some regarded them as a simplified interpretation of data when the microglial development and function had not been fully elucidated [47]. Interestingly, microglial polarization was observed in the cerebral haemorrhagic stroke, indicating an involvement of the microglia in producing proinflammatory or anti-inflammatory factors. Besides, the phagocytosis of cell debris and the potential crosstalk between microglia and T lymphocytes, neurons, astrocytes, and oligodendrocytes are also important pathologies after haemorrhagic stroke [48]. Additionally, in response to specific stimuli or with neuroinflammation, the microglia also have the capacity to damage and kill neurons. The uncontrolled microglial inflammatory response may result in the neuronal injury in neurodegenerative diseases, including Parkinson's disease (PD), Huntington's disease (HD), AD, and prion diseases, chronic traumatic encephalopathy, amyotrophic lateral sclerosis, and frontotemporal dementia [49]. Finally, although many mysteries exist regarding the effects and mechanisms of the microglia in neurological diseases, it shows the microglia as a promising future to treat neurological disorders, especially to cure neurodegeneration.

Increasing evidence has proved that PTE has regulatory effects on neurological disorders, especially neurodegeneration and stroke [18]. However, the modulatory roles of PTE in microglial activation and following injury have not been given enough attention. Neurodegenerative diseases have strong connections with microglial activation [50, 51]. A recent study compared the protective effects of PTE and its structural analogues on neurodegenerative diseases. It evaluates activities of these analogues on the LPS-induced release of proinflammatory factors, containing NO, iNOS, IL-6, IL-1*β*, and TNF-*α*, in BV-2 cells, and further involvement of the MAPKs (ERK1/2, JNK, and p38) and NF-*κ*B signalling pathways [28]. Also, PTE was proved to attenuate the neuroinflammatory response induced by amyloid-*β* in BV-2 microglia through inhibiting the NLRP3/caspase-1 inflammasome pathway [26], indicating a therapeutic effect of PTE in AD. However, the interaction of microglia and released proinflammatory factors with neurons were not entirely elucidated. Consistent with the reported evidence, we found that LPS induced BV-2 activation, evidenced by the increased release of IL-6 and TNF-*α*, and further PTE treatment attenuated the level of TNF-*α* and IL-6 in the media of BV-2 microglia in a dose-dependent manner. Besides, coculturing with LPS-activated BV-2 microglia cells, rather than BV-2 or LPS alone, induced the shrinkage, floating, and decreased viability of SH-SY5Y. Obviously, PTE treatment improved the SH-SY5Y viability, attenuated the BV-2 viability, and decreased LDH release in this coculture system. Similarly, PTE alleviated apoptosis and oxidative stress of SH-SY5Y cells induced by coculturing with LPS-activated BV-2 microglia. These may indicate an interaction between activated BV-2 and SH-SY5Y cells via proinflammatory factors, which induces apoptosis and oxidative injury of SH-SY5Y, and PTE could inhibit the activation of BV-2 microglia and subsequent generation of proinflammatory factors, thus presenting a neuronal protective effect in neurodegenerative diseases and stroke.

SIRT-1 is an evolutionarily conserved protein belonging to the sirtuin family of NAD (+)-dependent deacetylases [30]. For a long time, SIRT-1 has been considered to be correlated with neural development and antiaging in mammals, offering significant potential as an effective treatment strategy for neurodegenerative diseases [29, 31]. Recently, SIRT-1 has been reported to present protective effects on neurological diseases including AD, PD, motor neuron diseases, depression, cerebral ischemia, and SAH, which may relate to its regulatory functions in metabolism, stress resistance, inflammation, oxidative stress, and genomic stability [30, 31, 33, 36]. Besides, in the CNS, especially in the hypothalamus, SIRT-1 exerts pivotal roles in modulating the circadian rhythm and systemic energy homeostasis [30]. Importantly, accumulating evidences have shown that brain SIRT-1 plays neuroprotective roles in the context of neurodegenerative disorders and cerebral ischemia [30]. Studies have reported that increased SIRT-1 expression is related to reduced LPS-induced synaptic dysfunctions, suggesting a potential intervention for oxidative stress-related neurodegenerative diseases [52]. Also, studies have shown that activating SIRT-1 alleviated the SAH injury primarily by inhibiting the TLR4 [34] and NF-*κ*B signalling pathway [36]. As to the relationship between microglia and SIRT-1, it was reported that SIRT-1 is decreased with the senescence of microglia and the SIRT-1 deficiency in microglia leads to memory deficits through IL-1*β* upregulation [32]. Another in vivo study showed that SIRT-1 promotes functional recovery and neuronal survival via attenuating microglial inflammation after spinal cord injury [35]. Therefore, it could be speculated that SIRT-1 is an intracellular modulator of microglia-mediated inflammation. Multiple studies have revealed that PTE could activate SIRT-1 signalling in hepatic [37], myocardial [38], and skeletal muscle cells [39]. Interestingly, a study reported that a two-month diet of PTE or its analogue resveratrol did not increase SIRT-1 expression or the downstream signalling activation, whereas it increased peroxisome proliferator-activated receptor (PPAR) *α* expression, and thus modulated cellular stress, inflammation, and AD pathology [44]. However, another study suggested that resveratrol-related SIRT-1 activation alleviates cerebral ischemia reperfusion injury by inhibiting proinflammatory cytokine, reducing oxidative stress and apoptosis, and may protect against AD by inhibiting amyloid-*β* fibril formation, antiamyloidogenic effect, and delaying cognitive decline [12]. The effect of PTE on SIRT-1 activation in the neural cells is still undocumented. In this study, PTE treatment significantly increased the expression of SIRT-1, decreased the acetylation of NF-*κ*B subunit p65 in BV-2 microglia, and thus alleviated LPS-induced release of TNF-*α* and IL-6, which further attenuated apoptosis and oxidative injury of cocultured SH-SY5Y neuronal cells. Furthermore, the regulatory effect on SIRT-1 and ac-p65 of PTE were reversed by SIRT-1 inhibitor EX527, as well as the neuroprotective effects, as indicated by the increased apoptosis and oxidative markers. These suggest that the effects of PTE on reducing microglial inflammation and subsequent neuronal oxidative stress and apoptosis are mediated by SIRT-1/NF-*κ*B signalling pathways.

## 5. Conclusions

**Figure 6:**
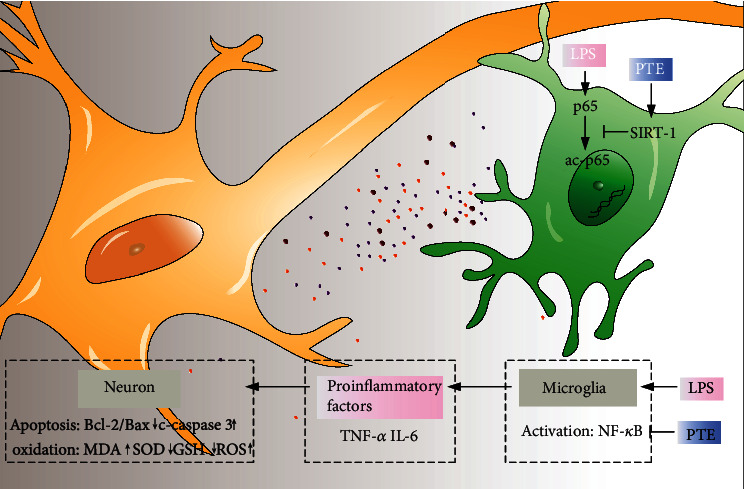
PTE attenuates microglial inflammation and neuronal apoptosis and oxidative stress via SIRT-1 pathway. PTE, pterostilbene. LPS, lipopolysaccharide. ROS, reactive oxygen species. MDA, malondialdehyde. SOD, superoxide dismutase. GSH, glutathione.

In summary, PTE reduced LPS-induced release of inflammatory factors, IL-6 and TNF-*α*, in BV-2 microglia, and thus attenuated oxidative stress and apoptosis in cocultured SH-SY5Y neural cells. Additionally, PTE mediated the downregulation of NF-*κ*B subunit p65 acetylation, which was likely due to increased SIRT-1 expression. This indicates that PTE treatment inhibited the NF-*κ*B pathway and might induce direct interactions between SIRT-1 and NF-*κ*B by stimulating SIRT-1 signalling, which then led to inhibition of LPS-induced microglial activation and subsequent inflammatory response, and thus attenuated the neuronal apoptosis and oxidative injury (Figure 6). These findings suggest PTE as a promising therapy of inflammation and oxidation-related diseases in the CNS, including neurodegenerative diseases and stroke, and provide a microglia-centric view of anti-inflammation and neuroprotection.

## Data Availability

The data generated during the study are available from the corresponding author by request.
